# Development of risk factor-based scoring system for detection of hypervirulent *Klebsiella pneumoniae* bloodstream infections

**DOI:** 10.1186/s13099-020-00374-5

**Published:** 2020-07-09

**Authors:** Atsushi Togawa, Michinobu Yoshimura, Chiemi Tokushige, Akira Matsunaga, Tohru Takata, Yasushi Takamatsu

**Affiliations:** 1 Department of Medical Oncology, Hematology, and Infectious Diseases, Fukuoka University Hospital, 7-45-1 Nanakuma, Jonan, Fukuoka, 8140180 Japan; 2 Department of Infection Prevention and Control, Fukuoka University Hospital, 7-45-1 Nanakuma, Jonan, Fukuoka, 8140180 Japan; 3 Department of Laboratory Medicine, Fukuoka University Hospital, 7-45-1 Nanakuma, Jonan, Fukuoka, 8140180 Japan; 4 Present Address: Department of Developmental Medicine, Research Institute, Osaka Women’s and Children’s Hospital, Osaka, Japan

**Keywords:** Hypervirulent *Klebsiella pneumoniae*, Bloodstream infection, Hypermucoviscosity, Capsular serotype, Scoring system

## Abstract

**Background:**

Hypervirulent *Klebsiella pneumoniae* (HVKp) infections have distinct clinical manifestations from classical *K. pneumoniae* infections. The hallmark of HVKp infections are liver abscess formation and metastatic infections. Due to the severe sequelae of these complications, method to identify patients at-risk of HVKp infections should be developed.

**Results:**

A retrospective cohort study of 222 patients with *K. pneumoniae* bloodstream infections (BSIs) was performed. Patient demographics, clinical manifestations, and bacterial characteristics were investigated. Ten cases of liver abscesses were identified. Characteristics such as community-onset BSIs, hypermucoviscosity phenotype, and capsular serotype K1 were identified as risk factors for HVKp infections. A scoring system was developed based on the risk factors. The area under the receiver operating characteristic curve for the scoring system was 0.90. A score of ≥ 2 points provided sensitivity and specificity of 0.70 and 0.94, respectively.

**Conclusions:**

Simple scoring system was developed for the diagnosis of HVKp infections. The system allows early identification of patients with *K. pneumoniae* BSIs in whom hypervirulent infections should be evaluated. Prospective evaluation is expected.

## Background

*Klebsiella pneumoniae* is a common hospital-acquired pathogen, causing classical *K. pneumoniae* (CKp) infections such as pneumonia, urinary tract infection, intra-abdominal infection and bacteremia in immunocompromised patients [1]. In 1986, physicians from Taiwan reported seven cases of pyogenic liver abscess associated with septic endophthalmitis by *K. pneumoniae* [2]. A similar case was reported from Spain later [3]. Since then the number of reported cases of pyogenic liver abscesses and associated metastatic infections, such as endophthalmitis, meningitis, and necrotizing fasciitis, by *K. pneumoniae* is rising [4–6]. These symptoms have never been observed in CKp infections.

The clinical and bacterial characteristics of these invasive syndromes, named as hypervirulent *K. pneumoniae* (HVKp) infections, have been extensively studied [6–8]. The distinctive characters of HVKp infections could be summarized as follows; (1) they are predominantly reported from Taiwan and Southeast Asia compared to the worldwide distribution of CKp infections; (2) common types of HVKp infections almost always include pyogenic liver abscess and, occasionally, other metastatic infections; (3) the typical mode of HVKp infections is community-onset infection in both immunocompetent and immunocompromised patients, compared to CKp which usually causes nosocomial infection in immunocompromised patients; (4) strains isolated from HVKp infections possess the hypermucoviscosity phenotype, which reflects the hyperproduction of capsules and can easily be detected by the string test [9], and the preferential distribution of capsular serotypes K1 and K2 [10].

Risk factors for HVKp infections in patients with *K. pneumoniae* bloodstream infections (BSIs) has been studied. Li et al. showed that diabetes mellitus and community-acquired BSIs are independent risk factors for HVKp BSIs [11]. Harada et al. reported that patients with HVKp infection had higher proportions of diabetes mellitus, and their infections had significantly higher propensity to liver abscess formation among Japanese patients with *K. pneumoniae* BSIs [12]. In these studies, the definitions of HVKp infections were based on molecular assays, in which the detection of virulence-associated genes, such as *rmpA*, *iroBCDN*, *iucABCD*, and *iutA*, was a prerequisite for defining them as HVKp.

Even though the diagnostic criteria of HVKp infections has still not been formally established [7], definition of HVKp infections should be based on clinical manifestations rather than microbiological analyses, since molecular analyses of virulence genes do not seems to be feasible in many microbiology laboratories. For precise surveillance of the incidence and clinical spectrum of HVKp infections, proper diagnostic criteria should be established, and if microbiology methods were to be applied, they should be as simple as possible to be used as routine practice in microbiology laboratories. In addition, based on such criteria, methods to identify patients with *K. pneumoniae* BSIs who are at-risk of HVKp infections should be investigated.

In this study we enrolled patients with *K. pneumoniae* BSIs for 10-year period and analyzed the clinical and bacterial characteristics to clarify the risk factors for HVKp infections. Based on the identified risk factors we tried to develop a scoring system to identify patients with *K. pneumoniae* BSIs who are at-risk of HVKp infections.

## Results

222 unique episodes of *K. pneumoniae* BSIs were identified, of which 103 (46%) episodes were community-onset. Since community-onset infection is one of the hallmarks of HVKp infections, we compared clinical manifestations of community-onset BSIs with those of hospital-onset BSIs (Table 1). Comparing to hospital-onset BSIs, patients with community-onset BSIs were older (72.6 v. 64.4) and biliary tract diseases were more often observed as underlying disease (34.0% v. 14.3%). Five patients (4.9%) with community-onset BSIs had no underlying disease, whereas all patients with hospital-onset BSIs had underlying diseases. Most notably, liver abscess was observed only in patients with community-onset BSIs. No significant difference was observed in antibacterial treatments in both patient cohorts, and the 30-day survival was comparable (82.3% v. 84.6%).

**Table 1:** Clinical and bacterial characteristics of *K. pneumoniae* BSIs

Characteristics	Community-onset BSIs		Hospital-onset BSIs		p value*
	N	%	N	%	
Patient demographics					
Median age, years (range)	72.6	69.3–75.8	64.4	61.3–67.4	0.0004
Male gender	61	59.2	76	63.9	0.478
Underlying disease					
Biliary tract disease	35	34.0	17	14.3	0.0005
Diabetes mellitus	33	32.0	27	22.7	0.118
Malignancy					
Hematologic	5	4.9	19	16.0	0.008
Solid organ	43	41.8	37	31.1	0.099
Other	15	14.6	34	28.6	0.012
None	5	4.9	0	0	0.015
Site of infection					
Liver abscess	10	9.7	0	0	0.0005
Biliary tract infection	43	41.8	17	14.3	< 0.0001
Other intra-abdominal infection	5	4.9	6	5.0	0.949
Urinary tract infection	20	19.4	31	26.1	0.241
Pneumonia	10	9.7	12	10.1	0.926
Febrile neutropenia	3	2.9	22	18.5	0.0003
Other/unknown	14	13.6	31	26.1	0.021
Antimicrobial treatment					
Penicillins	21	20.4	28	23.5	0.574
Cephalosporins	44	42.7	37	31.1	0.073
Carbapenems	36	35.0	49	41.2	0.341
Other antimicrobials	0	0	3	2.5	0.105
None	1	1.0	4	3.4	0.231
30-day survival (n = 213)	79	82.3	99	84.6	0.648
Bacterial phenotypes					
Capsular serotype					
K1	10	9.7	5	4.2	0.103
K2	14	13.6	14	11.8	0.683
Non-K1/K2	79	76.7	100	84.0	0.168
Hypermucoviscosity	15	14.6	6	5.0	0.016
Capsule-related gene (n = 133)					
magA gene	6/52	11.5	5/81	6.2	0.273
rmpA gene	11/52	21.2	8/81	6.2	0.010
Antimicrobial resistance					
ESBL-producer	2	1.9	22	18.5	< 0.001
Carbapenem resistance	0	0	1	0.8	0.351

*Pearson’s χ^2^-test

Analyses of isolated strains from these *K. pneumoniae* infections revealed several distinct characters with community-onset BSIs. Hypermucoviscosity phenotype was more often observed in community-onset BSIs than in hospital-onset BSIs (14.6% v. 5.0%). When the expression of *rmpA* gene, which is associated with the production of capsules [13], was determined in 133 strains, the gene was preferentially expressed in strains isolated from patients with community-onset BSIs than with hospital-onset BSIs (21.2% v. 6.2%). In terms of antimicrobial resistance, ESBL-producing *K. pneumoniae* was detected in 1.9% and 18.5% of patients with community-onset and hospital-onset BSIs, respectively. Resistance to carbapenems was observed in only one case of hospital-onset BSI.

Since liver abscess formation is uniformly associated with HV *K. pneumoniae* infections, we checked the medical records to identify patients in whom abdominal computed tomography (CT) scans were performed. We looked for cases with abdominal CT scans available and excluded cases examined with abdominal echograms, since abdominal CT scan is the most reliable method to detect liver abscesses caused by *K. pneumoniae* [14, 15]. Among our patient cohort, 127 (57.2%) patients received abdominal CT scans irrespective of contrast-enhancement within 7 days after the onset of *K. pneumoniae* BSIs. Abdominal CT scans were preferentially performed in patients aged 65 or older (odds ratio (OR), 1.98; 95% confidence interval (CI) 1.12–3.51) or in cases of community-onset BSIs (OR, 2.70; 95% CI 1.55–4.70) by univariate analyses.

We found 10 cases of liver abscesses in patients checked by abdominal CT scans. Among these cases we found a case complicated by endophthalmitis and another case with vertebral osteomyelitis and epidural abscess. In the former case, onset of *K. pneumoniae* BSI and the endophthalmitis was at the same time. However, in the second case, lower back pain has developed after the onset of *K. pneumoniae* BSI, and the vertebral osteomyelitis and epidural abscess was diagnosed after 3 weeks from the onset of BSI by magnetic resonance imaging.

When cases with and without liver abscesses were compared, several differences in clinical manifestations and bacterial characteristics were found (Table 2). All cases of liver abscesses were observed in community-onset BSIs, which contrast to cases without liver abscesses in which 53% of BSI episodes were community-onset. Thirty-day mortality was not significantly different between cases with and without liver abscesses (30% v. 18%). Absence of any underlying disease was more often identified in cases with liver abscesses than in cases without liver abscesses (20% v. 3%). In terms of bacterial characteristics, K1 capsular serotype and hypermucoviscosity phenotype were more often observed in isolates from patients with liver abscesses than in isolates from patients without liver abscesses (50% v. 6% and 50% v. 10%, respectively).

**Table 2:** Factors affecting the liver abscess formation

Characteristics (N = 127)	Liver abscess (+) (N = 10)	Liver abscess (−) (N = 117)	OR, 95% CI	p value*
Patient demographics				
Male gender	7 (70)	73 (62)	1.41, 0.35–5.72	0.633
Age ≥ 65 years	8 (80)	86 (74)	1.44, 0.29–7.16	0.653
Community-onset BSIs	10 (100)	62 (53)	N/A	0.004
Underlying disease				
Biliary tract disease	2 (20)	29 (25)	0.76, 0.15–3.78	0.735
Diabetes mellitus	3 (30)	35 (30)	1.00, 0.25–4.11	0.996
Malignancy				
Hematologic	0 (0)	10 (9)	0	0.335
Solid organ	2 (20)	33 (28)	0.64, 0.13–3.15	0.577
Other	2 (20)	29 (25)	0.76, 0.15–3.78	0.735
None	2 (20)	3 (3)	9.5, 1.38–65.28	0.007
30-day mortality (n = 120)	3 (30)	20 (18)	0.68, 0.17–2.79	0.363
Bacterial phenotypes				
Capsular serotype K1	5 (50)	7 (6)	15.71, 3.66–67.40	< 0.0001
Capsular serotype K2	0 (0)	18 (16)	0	0.181
Hypermucoviscosity	5 (50)	12 (10)	8.75, 2.21–34.64	0.0004
ESBL-producer	0 (0)	8 (7)	0	0.393

*Pearson’s χ^2^-test

**Fig. 1:**
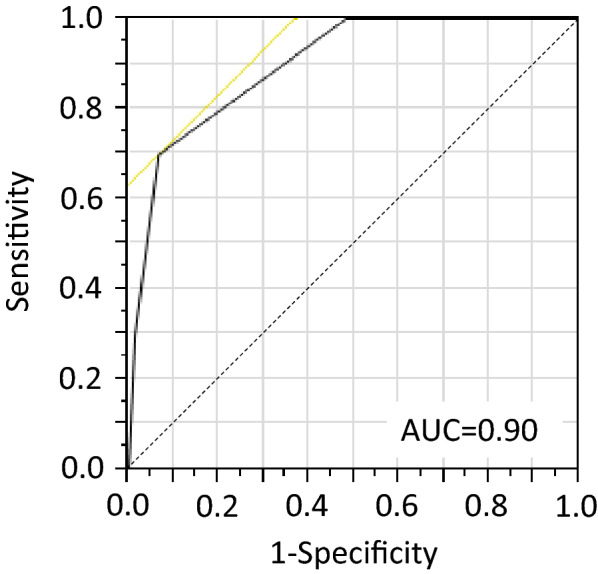
Receiver operator characteristic curve analysis for the scoring system

Based on these observations, we performed multivariate analyses to identify risk factors for liver abscess formation (Table 3). When adjusted by age, sex, and underlying diseases, community-onset BSIs (p = 0.0005), K1 capsular serotype (p = 0.0003), and hypermucoviscosity phenotype (p = 0.01) were identified as independent risk factors for liver abscess formation. Next, we evaluated whether these risk factors could be used as scores to identify cases with liver abscess formation. Each risk factor was given one point, so that the total scores would be from zero to three points (Table 3). To determine the cut-off value for the identification of cases with liver abscesses, we performed receiver operating characteristic (ROC) curve analysis (Fig. 1). The area under ROC was 0.90, indicating that the analysis was highly accurate. The sensitivity and specificity for each score were shown in Table 4. The score of ≥ 2 points provided maximal Youden’s index with a sensitivity and specificity of 0.70 and 0.94, respectively.

**Table 3:** Risk factors for liver abscess formation

Factors	aOR, 95% CI^a^	p value^†^	Points
Community-onset BSIs	N/A	0.0005	1
Capsular serotype K1	26.47, 4.56–206.38	0.0003	1
Hypermucoviscosity phenotype	9.59, 1.77–51.20	0.010	1

^†^Pearson’s χ^2^-test

^a^Adjusted by age, sex, and underlying diseases

**Table 4:** Accuracy of the proposed scoring system for the diagnosis of HV *K. pneumoniae* infection

Score	Liver abscess (+)	Liver abscess (−)	Sensitivity	Specificity	Youden’s index (J)
≥ 0	10	212	1	0	0
≥ 1	10	101	1	0.52	0.52
≥ 2	7	12	0.70	0.94	0.64
3	3	3	0.30	0.99	0.29
Number in sample	10	212			

## Discussion

The definition of HVKp is not formally defined. The reason may be that the precise meaning of the term “hypervirulent” in this context is ambiguous. In our study, we determined the definition of HVKp infections as “infections by *K. pneumoniae* complicated by liver abscess formation” with the assumption that CKp infections could never lead to liver abscess formation. This definition may contradict to literatures in which bacterial phenomenon of hypermucoviscosity was used as the definition for HVKp infections. Our idea is in line with the review by Catalán-Nájera et al., which suggested that the hypermucoviscosity and the hypervirulence are two different phenotypes that should not be used synonymously [16].

One of the most notorious characteristics of HVKp infections is its tendency to cause metastatic infections such as endophthalmitis. The treatment of bacterial endophthalmitis is difficult, and total loss of vision may ensue in the affected eyes [17]. Furthermore, even if metastatic infections are not obvious in early stage of infections, they may become apparent in later stage of infections as was noticed in our case. Thus, to recognize the possibility of these complications, it should be important to promptly identify cases with the risk for these complications. For this purpose, identification of patients at-risk of liver abscess formation should be sufficient, since metastatic infections are accompanied by liver abscess formation in HVKp infections.

Through the analyses of the patient cohort of *K. pneumoniae* BSIs, we identified three factors, namely, community-onset BSIs, the hypermucoviscosity phenotype, and capsular serotype K1 of the isolated strains, as risk factors for liver abscess formation. By utilizing these risk factors as points, we developed a scoring system. The system seems to be accurate with the AUROC of 0.90, and the maximal Youden’s index was obtained when the score was ≥ 2 points. This scoring system might be a useful tool to identify patients at-risk of HVKp infections, and prospective evaluation of this system should be performed.

Among the points for the scoring system, the identification of community-onset infection and the hypermucoviscosity phenotype should be a straightforward process. The maneuver of the string test is simple and should be available to almost all clinical microbiology laboratories. Compared to these factors, identification of *K. pneumoniae* capsular serotype is not an easy task. Quellung method, which is the traditional method for the identification of *K. pneumoniae* capsular serotype [18], is laborious and outdated. Other method using antisera [19] could be done in reference laboratories or research institutions but would be difficult to perform as routine practices in most laboratories. PCR method is usually employed to determine the capsular serotype but is laborious and not logistically applicable to all hospital settings. Compared to these methods, the immunochromatographic method [20] is very simple to perform and is possible to obtain the result within a few minutes. Even though the commercial product of this method is currently approved only in Taiwan for the clinical diagnosis of HVKp infections, and it can detect only capsular serotypes K1 and K2, it is an attractive tool to quickly identify capsular serotype K1. When this method is available, by using isolated colonies the clinical microbiologists can detect both the hypermucoviscosity phenotype and capsular serotype K1 or K2 within a short time and inform the clinicians promptly. In our experience, the whole process of calculating the scores, including the chart review and microbiology analyses, and providing any recommendation regarding the possible complications associated with HVKp infections could be completed within 1 h.

Our study has some notable limitations. First, the study was performed in a single-center setting, so that some important risk factors for HVKp infections may be missed. In addition, the fact that all patients except one were of Japanese ethnicity may have biased the types of risk factors in an ethnicity-related way. Second, not all patients with *K. pneumoniae* BSIs received CT scans, so that some cases of liver abscess may be missing. In this study the decision to perform CT scans was at the discretion of attending physicians. However, through the investigation of patient charts who did not receive CT scans, the risk of the development of liver abscesses appeared to be minimal in these patients, and we assume that all cases of liver abscess could be identified in our study. Third, we did not pick up the genetic characteristics of isolates as risk factors for HV infections. *RmpA* gene was preferentially detected in isolates from community-onset BSIs, and it could be an additional risk factor for HV infection. However, since the purpose of this study was to develop simple scoring system which can be performed in most microbiology laboratories as routine practices, the exclusion of genetic analyses should be justified.

## Conclusions

We analyzed the patient cohort of *K. pneumoniae* BSIs and identified three factors, community-onset infection, hypermucoviscosity phenotype, and capsular serotype K1, as risk factors for HVKp infections. Based on the results we developed a simple scoring system to identify HVKp infections with the sensitivity of 70% and the specificity of 94% when scores are ≥ 2 points. This scoring system may enable every clinician to efficiently identify patients with *K. pneumoniae* BSIs who are at risk for HVKp infections and its associated metastatic complications.

## Methods

### Study design and case definition

The study was conducted in a 900-bed tertiary-care university hospital from April 2009 to May 2019. Episodes of *K. pneumoniae* BSIs were identified from the records of the clinical microbiology laboratory. If multiple episodes of *K. pneumoniae* BSIs were identified in a patient, only the first episode was involved in the study. All patients except one were of Japanese ethnicity. Clinical characteristics were collected from the electronic health record of each patient. *K. pneumoniae* BSIs identified ≤ 72 h or > 72 h after the admission to the hospital were designated as community-onset or hospital-onset infections, respectively. Hypervirulent *K. pneumoniae* infections was defined as infections by *K. pneumoniae* associated with liver abscess formation. The study was approved by the institutional review board (No. 18-11-03).

### Microbiology

Blood isolates were identified as *K. pneumoniae* by Vitek II system (bioMerieux, Japan) and/or MALDI TOF-MS (Bruker, Japan). Isolates were frozen at − 80 °C until analysis. Isolates were cultured on Columbia agar with 5% sheep blood (Nippon Becton-Dickinson, Japan) overnight. String tests were performed on all isolates. The string test was diagnosed as positive when a bacterial inoculation loop produced a viscous string of > 5 mm length by stretching bacterial colonies on agar plates. *K. pneumoniae* strains with a positive string test were determined as having the hypermucoviscosity phenotype.

### Capsular serotype analysis

For isolates from 2009 to 2016, presence of capsular polysaccharide K1 and K2 genes was determined by polymerase chain reaction (PCR) as described previously [10]. In 2017, PCR method was switched to immunochromatography method by utilizing the rapid testing cassette for the detection of capsular serotypes K1 and K2 (KeMyth Biotech Co., Taiwan) [20]. By using the rapid testing cassettes, it is possible to identify the capsular serotypes as K1, K2, or non-K1/K2 within several minutes. Before switching the method, we evaluated the performance of the rapid testing cassettes by comparing the results of PCR with the rapid testing cassette to identify capsular serotype K1 and K2. Analysis of 23 isolates showed that the test results by rapid testing cassettes matched completely with the test results by PCR (unpublished data).

### Statistics

Patient demographics and laboratory data were processed by JMP software ver. 10 (SAS Institute Japan, Tokyo, Japan). The χ^2^ test was used to compare categorical variables. Student’s t-test was used to compare numerical variables. Logistic regression analyses were used to determine which risk factors were statistically significant for each category. Receiver operating characteristic curve was used to determine the cut-off value for the assessment of HV *K. pneumoniae* infections based on the scoring of each risk factor.

## Data Availability

The datasets used and/or analysed during the current study are available from the corresponding author on reasonable request.
